# Editorial: AI and Multi-Omics for Rare Diseases: Challenges, Advances and Perspectives

**DOI:** 10.3389/fmolb.2021.719978

**Published:** 2021-06-30

**Authors:** Silvia Bottini, Frank Emmert-Streib, Leonardo Franco

**Affiliations:** ^1^ Center of Modeling, Simulation and Interactions, Université Côte d’Azur, Nice, France; ^2^ Predictive Society and Data Analytics Lab, Faculty of Information Technology and Communication Sciences, Tampere University, Tampere, Finland; ^3^ Computer Science Department, Malaga University, Malaga, Spain

**Keywords:** rare disease, artificial inteligence, bioinformatic, multi-omic, diagnosis

A rare disease (RD) is any disease that affects a small percentage of the population. In Europe a disease or disorder is defined as rare when it affects less than 1 in 2,000 citizens. There are more than 7,000 RDs worldwide. Although individually rare, collectively RDs are estimated to affect 350 million people globally. Most rare diseases are genetic and are present throughout a person’s entire life, even if symptoms do not immediately appear. RDs are characterized by each having a wide diversity of symptoms, which can vary from patient to patient. Symptoms of RDs can also appear to be similar to those of common diseases. These factors mean that RDs can often be misdiagnosed. According to the Global Genes organization, 8 out of 10 RDs are caused by a faulty gene and approximately 75% affect children, yet it takes an average of 4.8 years to arrive at an accurate diagnosis. This is part of the reason for 30% of children with RDs not living to see their fifth birthday. There are numerous challenges and issues that need to be addressed, ranging from technical to theoretical points of view, such as the small number of patients (often children), the heterogeneity of the disease, and the limited amount of national/international data resources.

The development of new technologies, such as genomic analysis by means of next generation sequencing (NGS) and other “omics technologies,” has boosted the molecular understanding and diagnosis of RDs. However, there is a growing need to develop new methods to integrate multi-omics data from different technologies. Furthermore, the ability of AI technologies to integrate and analyze data from different sources (e.g., multi-omics, patient registries) can be used to overcome further challenges, such as low diagnostic rates, reduced number of patients, and geographical dispersion. Ultimately AI-mediated knowledge could significantly boost therapy development for RDs.

Owed to this advance, our Research Topic has collected contributors that describe the current methodologies, applications, challenges facing RD diagnosis, practical insight into improving data analysis techniques as well as advances in bioinformatics and AI approaches for biomedical research in RD. This research topic garnered five articles, including four comprehensive reviews and one original research article. These articles span multiple type of rare disease from mitochondrial diseases to neuromuscular disorders and hepatocellular carcinoma. Notably, they present a multitude of approaches not only bioinformatics and AI, and were contributed by academic institutes and hospitals engaged in rare diseases, demonstrating the great interest and applications in this hot area.

To brighten the plethora of multi-omics integration tools that the community of computational biologists and bioinformaticians has developed, our team performed a comprehensive review, with a special sight in mitochondrial diseases applications, proposing a novel data-driven classification (Labory et al.). We point out that mitochondrial diseases, as well as other rare diseases, are extremely heterogeneous both clinically and genetically, with a broad range of age onset and very different symptoms, that makes their diagnosis very challenging.

About 80% of rare diseases have genetics origins, meaning that they are caused by pathogenic variants affecting the genome. The advent of whole-exome sequencing and whole-genome sequencing has accelerated the identification of variants on previously unknown disease genes. Although these technologies are mainstays in Mendelian disease diagnosis, their success rate for detecting causal variants is far from complete, ranging from 25 to 50%. Several variants remain as variants of unknown significance or they are missed due to the inability to prioritize them such as intronic or non-coding variants. Recently, the employ of RNA sequencing has been proposed. The review of Schlieben et al. provides an overview of the methods used for RNA sequencing and illustrate how these can improve the diagnostic yield of rare diseases.

An additional layer of complexity for RD diagnosis is added when considering that the surrounding environment of an individual also plays a determinant role in disease development and progression. This can have consequences on the epigenome that includes the mechanisms by which changes in gene expression occur without changing the DNA sequence. The review of Brasil et al. discuss how the interplay between genetics and epigenetics should be considered when addressing the etiology of RDs and the fundamental role played by AI tools for diagnosis, disease characterization, and therapeutic approaches in RDs.

Another approach to face RD diagnosis challenges is to design algorithms and approaches that combine phenotype and gene information to facilitate diagnosis and improve treatment. Taking advantage of several existing phenotypic databases, Diaz-Santiago et al. developed a novel approach that provide insight into the cellular functions and phenotypic patterns underlying neuromuscular disorders.

Finally, the review by Li et al. provides a comprehensive, up-to-date overview of integrative approaches used to improve the underlying mechanisms of combined systemic and locoregional therapies in the treatment of advanced hepatocellular carcinoma, commenting on both their current status and future direction.

Despite the fact that the application of AI algorithms in RD is still in its infancy stage, it has opened the door to a more faithfully understanding of the complex aspects of RD physiology, pathology, and treatment. Thereby, it is time to apply these powerful algorithms to gain a better understanding, diagnosis and treatment of RD and the underlying biology. Much remains to be learned and developed, and we hope this Research Topic has captured the essence of this field and will inspire future innovations and breakthroughs in both the understanding and diagnosis of RD.

